# Lung ultrasound in the COVID-19 pandemic

**DOI:** 10.1136/postgradmedj-2020-138137

**Published:** 2020-09-07

**Authors:** Karl Jackson, Robert Butler, Avinash Aujayeb

**Affiliations:** Respiratory and Acute Medicine Department, Northumbria HealthCare NHS Foundation Trust, Newcastle NE23 6NZ, United Kingdom; Respiratory and Acute Medicine Department, Northumbria HealthCare NHS Foundation Trust, Newcastle NE23 6NZ, United Kingdom; Respiratory and Acute Medicine Department, Northumbria HealthCare NHS Foundation Trust, Newcastle NE23 6NZ, United Kingdom

**Keywords:** Ultrasonography, Thoracic medicine, Adult thoracic medicine, Respiratory infections

## Abstract

Lung ultrasound has been described for over a decade and international protocols exist for its application. It is a controversial area among pulmonologists and has had more uptake with emergency as well as intensive care physicians. We discuss the basics and evidence behind the use of lung ultrasound in respiratory failure, and what role we see it playing in the current 2019 novel coronavirus pandemic.

## COVID-19 ERA

The 2019 novel coronavirus (SARS-Cov-2), the pathogen responsible for the new disease 2019 novel coronavirus (COVID-19) has resulted in a worldwide pandemic. At the time of writing, there have been 3 175 207 cases and 224 172 deaths.^1^ The virus has ushered in a new era of healthcare with nationwide organisations such as the National Health Service in the United Kingdom (UK) reorganising and restructuring services to provide for acutely ill patients.^2^

A review of 16 749 hospitalised UK patients with COVID-19 using the International Severe Acute Respiratory and emerging Infections Consortium (ISARIC) WHO Clinical Characterisation Protocol found the most common symptoms to be cough (70%), fever (69%) and shortness of breath (65%), all part of a syndrome of acute respiratory distress.3 49% of patients were discharged alive, 33% had died and 17% were continuing to receive care at date of reporting; 17% required admission to high dependency or intensive care units for treatment of respiratory failure.^3^

Many patients with COVID-19 present with relatively preserved lung compliance and a severely compromised pulmonary gas exchange which leads to severe hypoxaemia. By increasing their minute ventilation (compensatory ventilatory response to hypoxaemia), patients develop severe hypocapnia.^4^

The pathophysiological explanation for respiratory failure in COVID-19 is still being studied but it is likely that diffuse alveolar damage with interstitial thickening, varying degrees of atelectasis and consolidation contributes.^5^

Case series have described bilateral chest radiograph abnormalities such as diffuse interstitial changes in over 84% of patients with the detection of changes approaching 100% with the use of CT.^6^

## STETHOSCOPE AND THE RESPIRATORY EXAMINATION

The above respiratory symptoms require a full assessment, and the first step is normally a clinical examination of the respiratory system, in which the stethoscope plays a major role.

In 1816, a French physician, René Laennec, invented the stethoscope and was also the first to describe the terms rales, rhonchi, crepitance, and egophony which are associated with specific respiratory conditions.^7^ Acquiring and wearing a stethoscope has become a rite of passage for doctors and has formed an integral part of the diagnostic pathway.^8 9^ The respiratory examination comprises four basic tenets: inspection, palpation, percussion and auscultation. Auscultation requires the stethoscope to be applied to the front and back of chest, as well as under the axillae.^8 9^ However, the signs are neither specific nor sensitive. The main sign in respiratory failure syndromes is crackles.

Crackles on auscultation have a sensitivity of 19–67% and a specificity of 36–96%, a positive likelihood ratio of 2.3 and a negative likelihood ration of 0.8.^10^ Thus, their use in ruling pneumonia in or out is limited as their presence or absence only slightly changes the initial diagnosis (poor inter-observer reliability, 72% agreement, kappa value 0.41).^11^ Lovrenski *et al* replicated similar findings in children with suspected pneumonia.^12^

Given the high incidence of interstitial changes and respiratory failure in COVID-19 and the risk of cross-infection when taking a stethoscope from patient to patient, should lung ultrasound have an increased and complementary role to play in the diagnostic pathway?^13^

## LUNG ULTRASOUND IN RESPIRATORY FAILURE

Lung ultrasonography (LUS) in acute respiratory failure has been described for over a decade. A 2008 single centre study (Bedside Lung Ultrasound in Emergency [BLUE]) of LUS at the bedside showed an accuracy of 90.5% in diagnosing the cause of acute respiratory failure in critically ill patients.^14^ The methods were standardised and reproducible and consensus guidance has since been produced.^15^ The BLUE protocol also comprises the assessment of the deep venous system, but the discussion of this aspect is beyond the scope of this article. The BLUE protocol has higher sensitivity, specificity and diagnostic accuracy for pleural effusions, alveolar consolidation and interstitial syndromes: this is described by Lichtenstein *et al* ^16^ and all the values for LUS are between 92% and 100%. We will concentrate on core LUS; the full BLUE protocol for LUS is beyond the scope of this article.

**Figure 1:**
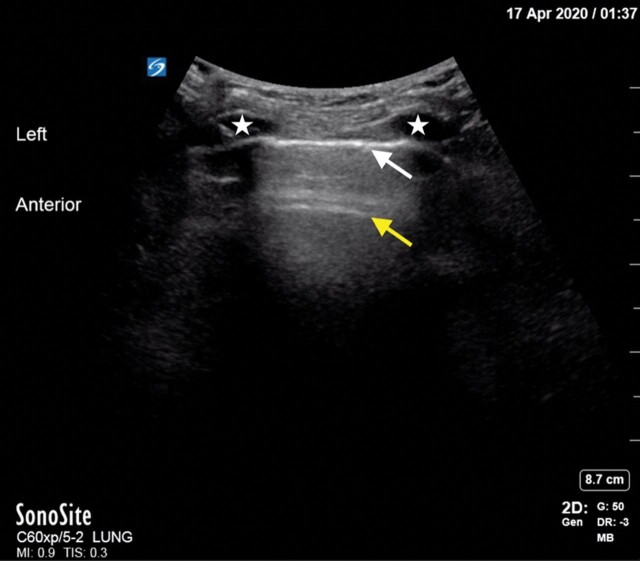
USS image showing pleural line (white arrow), A line (yellow arrow), ribs (white star).

**Figure 2:**
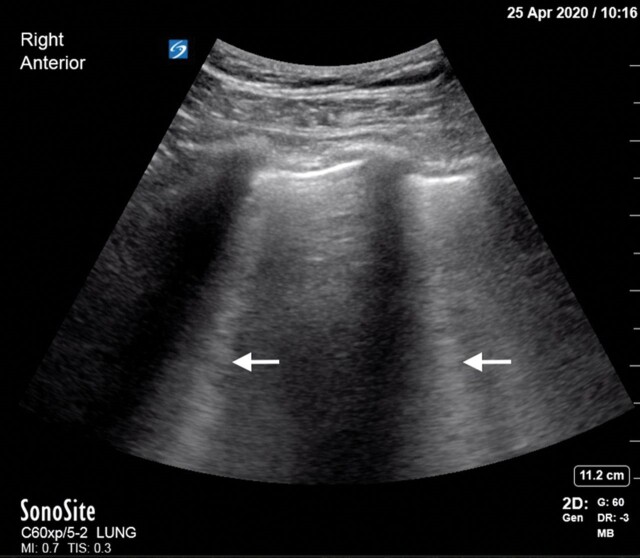
USS image showing multiple B lines.

**Figure 3:**
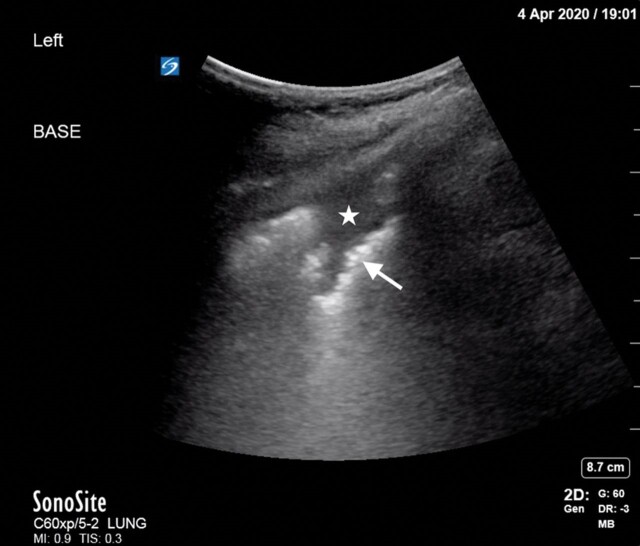
USS image showing shred sign with fractal line (white arrow), consolidated lung (white star).

**Figure 4:**
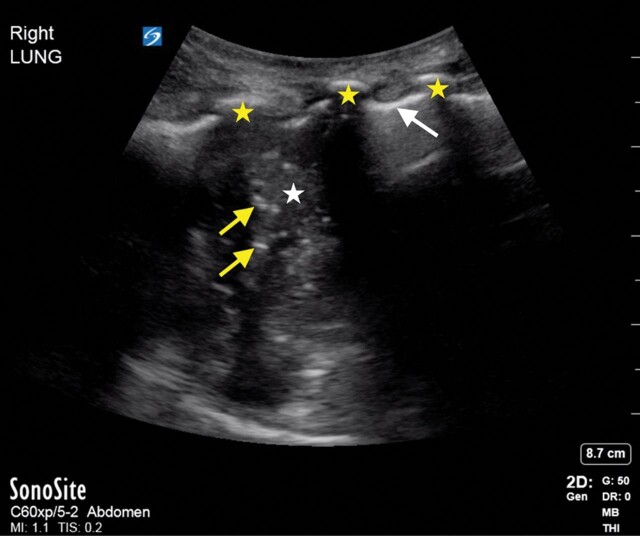
USS image showing right lung consolidation with hepatisation (white star), air bronchograms (yellow arrows); pleural line (white arrow), ribs (yellow star).

LUS requires the knowledge of the following signs:

1. Identification of the *pleural line* (figure 1, white arrow) and *lung sliding* (back and forth horizontal movement at the pleural line signifying the absence of pneumothorax).
2. The A*-ine* (figure 1, yellow line) (horizontal long path reverberation artefact of the pleural line); multiple A-lines can exist, spaced at regular intervals corresponding to the depth between chest wall and pleural line. A-lines and sliding suggest normally aerated lung; A lines without sliding suggest pneumothorax.
3. The *lung point* confirms pneumothorax.
4. The B-*lines* (figure 2, white arrow) (formerly known as lung rockets) indicate an interstitial syndrome and arise from the pleura. B-lines occur as three or more lines, erase A-lines and reach the far field of the ultrasound image. B lines move with lung sliding and essentially rule out obstructive lung disease and pneumothorax, while making pulmonary embolism less likely.
5. Consolidation is confirmed by the *fractal* or *shred sign* (figure 3, white arrow) as well as *hepatisation*. Consolidated lung tissue (figure 3, white star) appears as a subpleural hypoechoic region that may appear tissue-like or liver-like with an irregular (shredded) deep border (fractal line) abutting normally more aerated lung, which has echogenic artefacts.
6. Signs of effusion include the *quad* and *sinusoid signs* (the quad sign is a static sonographic sign consisting of four lines representing the pleura, rib, fluid, and lung). The sinusoid sign is an M-mode finding indicating respiratory movement of atelectatic lung in a sinusoidal pattern within surrounding pleural fluid. These two signs have a high sensitivity and specificity for pleural effusion. Figure 4 shows right lung consolidation with hepatisation (white star), air bronchograms (yellow arrows); pleural line (white arrow), ribs (yellow star).

**Figure 5:**
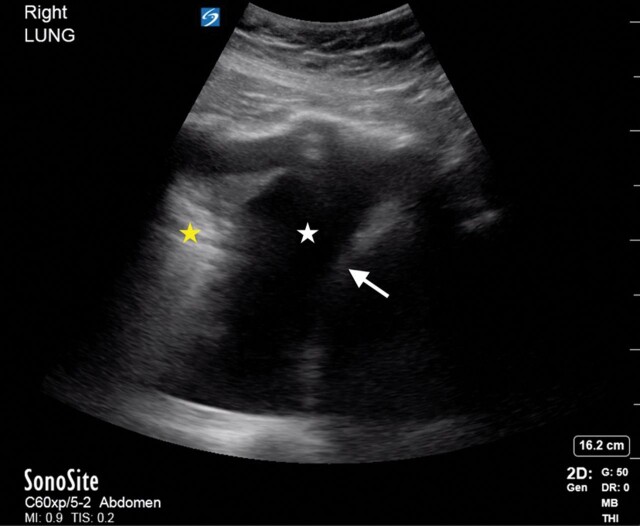
USS image showing right lung effusion (white star), atelectatic lung (yellow star) and diaphragm (white arrow).

Figure 5 shows right pleural effusion (white star), atelectatic lung (yellow star) and diaphragm (white arrow)

In the UK, Focused Acute Medicine Ultrasound (FAMUS) is the point of care ultrasound (POCUS) standard endorsed by the Society of Acute Medicine for Acute Physicians as a specialist skill^17^; Focused Ultrasound in Intensive Care (FUSIC) is the POCUS standard for the Intensive Care Society.^18^ Both offer ultrasound training targeted to prompt assessment and diagnosis of the acutely unwell adult patient.

## ROLE OF IMAGING AND LUNG ULTRASOUND IN COVID-19 PATHWAY

If a patient presents to hospital, the traditional pathway dictates a full history and examination, followed by targeted tests. In COVID-19, the gold standard for the diagnosis of COVID-19 is the positivity of a swab through reverse-transcription PCR (RT-PCR) but there can be a delay in that particular result.^19^ An initial negative result then requires a risk assessment as to whether the patient should be considered as high index clinical suspicion of COVID-19. Imaging can assist with further risk stratification. Chest radiography (CXR), using a template classification system produced by British Society of Thoracic Imaging (BSTI)^20 21^ groups patients into one of the four groups based on their CXR: COVID classic/probable, COVID indeterminate, COVID normal and Non-COVID. Classic CXR changes of COVID-19 are well described as showing bilateral multi-lobar consolidation or infiltrative changes, with multiple peripheral air space opacities.^22^ CT scans have been used as first-line investigation but this has significant challenges in terms of infection control and burden of work on radiology departments.^19 23^ CT scan findings typically show a combination of ground glass opacities and peripheral consolidations and are highly sensitive (approaching 97% in some cases,^24^) although not completely specific.^25^ Hence, it has been suggested that CT not be used as a first-line diagnostic test, but is reserved for a patient with high clinical suspicion of COVID-19 and a negative swab result.^19 25^

COVID-19 causes clear and typical ultrasonographic patterns. B lines in COVID-19 occur in large numbers, both in separate and coalescent forms (light-beam patterns), and can give the appearance of a shining white lung. Irregularity of the pleural line, sub-pleural pulmonary consolidations and poor blood flow also occur in bilateral patchy clusters, and are mainly visible in the posterior and inferior areas.^26 27^

Poggiali *et al* found strong correlation between similar ultrasound findings and concurrent CT scans.^28^ 12 patients with COVID-19 underwent both LUS and CT scanning. All patients had diffuse B−lines with spared areas. Three had posterior subpleural consolidations and four had the appearance of organising pneumonias: bilateral patchy subpleural or peripheral consolidations. CT scans confirmed bilateral lung involvement with ground-glass opacities and consolidation changes in all patients.^28^

Volpicelli *et al* argue that there are ultrasonographic patterns that would suggest alternative diagnoses to COVID-19.^29^ A smooth pleural line with uniform and gravity-dependent B-lines suggests cardiogenic pulmonary oedema. Diffuse abnormalities of the pleural line and no patchy distribution are more likely in chronic diffuse interstitial pulmonary fibrosis. Isolated large lobar consolidation with or without effusion and with dynamic air bronchograms indicate bacterial pneumonias and large pleural effusion with atelectatic consolidations or echoic septations would suggest another infection as COVID-19 does not seem to be associated with complex pleural disease.^30^

**Figure 6:**
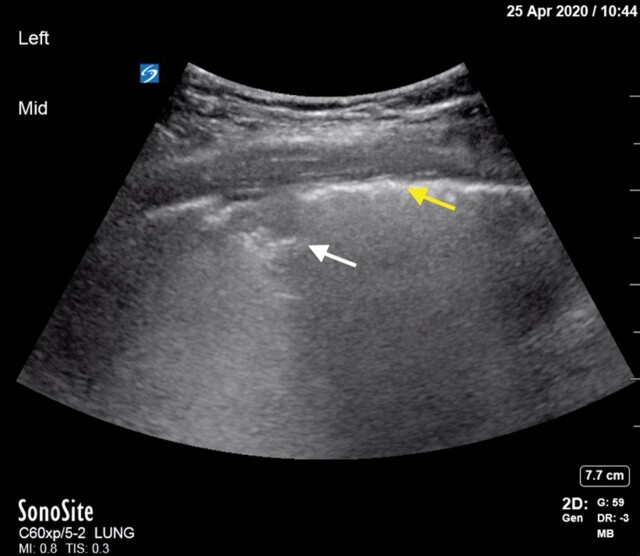
USS image of Covid-19 patient with sub pleural consolidation (white arrow) and irregular pleural line (yellow arrow).

**Figure 7:**
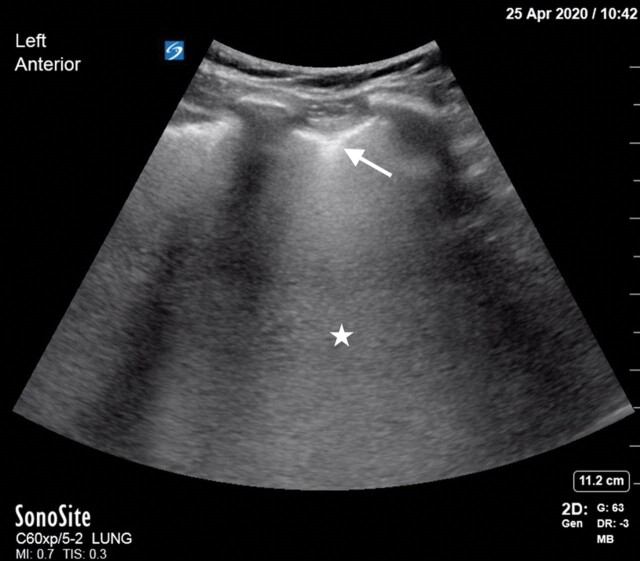
USS image of Covid-19 patient showing Covid with white lung (white star) and sub pleural consolidation (white arrow).

Soldati et al propose a standardised process for the use of LUS in COVID-19 patients,^31^ 60000 ultrasound frames from 30 patients were reviewed by an expert panel and then submitted to a biomedical engineer who then produced a grading system for LUS in COVID-19. In an attempt to achieve high inter-observer variability, the images produced by the engineer were re-submitted to the panel to evaluate and agree on the scoring system. A continuous pleural line with some A lines suggest moderate aeration and a score 0. An irregular pleural line with some B lines suggest some loss of aeration and a score of 1. Score 2 due to severe loss of aeration is suggested by a broken pleural line and small-to-large consolidated areas with associated areas of white below the consolidated area. Score 3 is attributed if the scanned area shows large dense consolidations which signify complete loss of aeration. The scores can be applied to every lung area scanned. Figure 6 shows the USS of a COVID-19 patient with subpleural consolidation (white arrow) and irregular pleural line (yellow arrow) and figure 7 shows white lung (white star) and subpleural consolidation (white arrow).

In the UK, the Intensive Care Society (ICS) and the Society for Acute Medicine (SAM) have jointly proposed guidance for the role of ultrasound in patients with suspected or proven COVID-19.^32^ It has also been suggested that pulmonary physiotherapists could help in expanding the use of LUS.^32^ Decontamination guidance has been produced for any machines used but the vast majority of (POCUS) machines are small, portable and routinely easily cleaned in between patients.^33^ A national service evaluation dataset has been created, and this uses the scoring system described by Soldati *et al.* ^31 34^

## POTENTIAL USES OF AND BARRIERS TO LUS IN COVID-19

There are currently no data to show for any outcomes for LUS in COVID-19. The setting up of national datasets as well as governance pathways provide this opportunity to study this area longitudinally and hopefully provide real-time analysis and any outcomes that may influence patient care.

Proposed potential uses of LUS are:

1. At triage: patients presenting with acute respiratory failure require rapid triage and prompt isolation in settings where resources may have already been stretched.^29 35^ A quick LUS (in experienced hands, the BLUE protocol can be done in less than 3 min^36^) can help with this.
2. In patients who are swab PCR negative and have an indeterminate CXR: the presence of the above classical changes of COVID-19 would suggest a false negative of the swab result and allow a firm diagnosis to be established. It must be remembered that LUS is an adjunct in the diagnostic pathway and is to be interpreted in light of all clinical findings and diagnostic considerations.
3. To determine ventilation strategies and post procedures in mechanically ventilated patients: In patients with bilateral and widespread multiple or coalescent B−line plus pleural irregularities, increased positive end-expiratory pressures may be beneficial; in patients with a relatively normal anterior lung, but posterior-lateral consolidation, prone ventilation may be tried.^37^ The resolution of pathological signs to an A line pattern also signifies disease improvement and may be used for monitoring.^38^ These two patterns were used by Italian and Chinese groups for day to day management of COVID-19 patients.^39 40^A Lung USS can also be useful at detecting, pneumothoraces in patients who are mechanically ventilated and guiding any ensuing procedures.^41^

However, LUS is a controversial topic among pulmonologists and emergency physicians and uptake has been traditionally low, with pulmonologists focussing more on the pleura than the lung.^42^ Some barriers to widespread use of POCUS are discussed below, and we expand on why those barriers should not exist.

1. The vast majority of physicians are also not trained at lung ultrasound and this might prove time consuming. We agree that there will be a learning curve with this, but with more physicians doing LUS and with physiotherapists weighing in with their specialised skills, more and more doctors will become adept at this.
2. Cost of procurement of the POCUS machines: over time, the machines have become more efficient, smaller and less costly.^33^ The availability of machines should not be a problem. We work in a large district general hospital in the North East of England, and we have six large machines and four handheld devices between the Emergency department, the Acute Medical Unit and the Respiratory unit. Locally, all non-urgent work has been stopped in line with national guidance^2^ and the ultra-sonographers and echocardiography technicians have all been offered training to perform lung ultrasound. This has had the effect of greatly increasing the number of people who can perform LUS and available machines. POCUS training and machines is already in place in low resource settings^43 44^ and there is no reason why LUS cannot be widely applied if and when COVID-19 reaches those settings.
3. Governance and storage of images: images from POCUS examinations are very rarely stored on a central database and therefore are not readily available for review for any potential refuting or confirmation of findings. A study in the United States over 20 years found five lawsuits associated with the use of POCUS. All were related to failure to perform POCUS or failure to perform one in a timely manner and there were no missing diagnoses or harm reported.^45^ Newer systems such as ones from the Butterfly Network^46^ can connect to existing picture archiving and communication systems (PACS) for storing and retrieving images on hospital networks.

Locally, in a large district general hospital in the North East of the United Kingdom, we have set up an ultrasound steering committee with a view to devise a strategy regarding lung USS. The key points are to:

- Devise a local policy as to where lung USS might play a role.
- Produce a training programme for interested physicians and physiotherapists.
- Ensure that any learning plan is incorporated into the practitioners professional learning and development plan.
- Ensure that the trainers have time in their job plans and funding set aside to develop the aforementioned programme.
- Ensure that the correct ultrasound machines are bought for all the department and that a single business plan and bid is submitted.
- Ensure that images can be uploaded to the local radiological online archive (PACS).

## CONCLUSION

COVID-19 has required a paradigm shift in healthcare utilisation. Imaging has a key role to play in the diagnostic pathway and LUS might play an important role. Lung ultrasound is a non-invasive, rapid, repeatable, and sensitive bedside method to detect a range of pulmonary pathologies. Whilst there is currently no clear data to show improved patient outcomes, the COVID-19 era poses unprecedented challenges but also learning opportunities. The current enthusiasm for LUS must be directed into strong controlled studies and descriptive analyses towards determining patient outcomes. LUS findings must also be taken in context of all other clinical and radiological.

**Main messages**

- COVID-19 patients have findings on lung USS which can be used to rule out COVID-19 alongside all other clinical and radiological parameters.
- There is a national drive in the UK to determine the role lung USS has in the diagnostic pathway of COVID-19.
- Lung USS needs wider uptake by healthcare practitioners and current pandemic provides a unique opportunity.

**Current research questions**

- What is the role of lung ultrasound in the triage of potential COVID-19 patients?
- What is the role of lung ultrasound in patients who are swab PCR negative and have an indeterminate CXR?
- What is the role of lung ultrasound in selecting ventilation strategies in the intensive care unit.

**Self-assessment questions**

1. Which one of the following statements about B-lines is TRUE?
  - B-lines do not extend all the way to the edge of the field without fading.
  - B-lines are reverberation artefacts arising from aerated and fluid parts of the lung.
  - B-lines are diagnostic of COVID-19.
  - B-lines show well-aerated lung.
2. Which one of the following statements is FALSE?
  - B-lines occur in COVID-19 patients.
  - B-lines occur in pulmonary oedema.
  - B-lines occur in acute respiratory distress syndromes.
  - B-lines occur in exacerbations of chronic obstructive pulmonary disease.
3. Which one of the following statements are TRUE?
  - Coalescent B−lines can occur in COVID-19 patients.
  - Irregularity of the pleural line can occur in COVID-19 patients.
  - Sub-pleural pulmonary consolidations can occur in COVID-19 patients.
  - Complex pleural disease is rare in the initial presentation of COVID-19 patients.
4. Which one of the following statements are TRUE?
  - CT scanning is not recommended as a first-line investigation in COVID-19.
  - CT scan findings have very high sensitivity and specificity for infection with COVID-19.
  - CT scanning of COVID-19 patients does not pose an infection control risk.
  - A CXR showing classic signs of COVID-19 requires a CT scan for confirmation of the diagnosis.
5. Which one of the following statements are TRUE?
  - There is clear evidence for the benefit of lung USS in the triage in COVID-19 patients.
  - There is clear evidence for the benefit of lung USS in COVID-19 patients who are swab PCR negative.
  - Uptake of lung USS among pulmonologists in the UK has traditionally been poor.
  - POCUS machines are large, bulky and difficult to treat.

**Key references**

1. Lichtenstein D, Meziere G. Relevance of lung ultrasound in the diagnosis of acute respiratory failure*: The BLUE Protocol. *Chest* 134(1):117–125, doi:10.1378/chest.07-2800.
2. https://www.ics.ac.uk/ICS/ICS/FUSIC/FUSIC_COVID-19.aspx (accessed 3.5.2020).
3. Volpicelli, G, Gargani, L. Sonographic signs and patterns of COVID-19 pneumonia. *Ultrasound J* 2020;12(22), doi:10.1186/s13089-020-00171-w.
4. Li X, Zeng W, Li X, *et al.* CT imaging changes of corona virus disease 2019 (COVID-19): a multi-center study in Southwest China. *J Transl Med* 2020;18:154, doi:10.1186/s12967-020-02324-w.
5. Soldati, G., Smargiassi, A., Inchingolo, R, *et al.* Proposal for international standardization of the use of lung ultrasound for patients with COVID-19. *J Ultrasound Med* 2020, doi:10.1002/jum.15285.

**Self-assessment answers**

(A) False (B) True (C) False (D) False
(A) True (B) True (C) True (D) False
(A) True (B) True (C) True (D) True
(A) True (B) False (C) False (D) False
(A) False (B) False (C) True (D) False
